# A semi-empirical risk panel to monitor epidemics: multi-faceted tool to assist healthcare and public health professionals

**DOI:** 10.3389/fpubh.2023.1307425

**Published:** 2024-01-08

**Authors:** Aida Perramon-Malavez, Mario Bravo, Víctor López de Rioja, Martí Català, Sergio Alonso, Enrique Álvarez-Lacalle, Daniel López, Antoni Soriano-Arandes, Clara Prats

**Affiliations:** ^1^Department of Physics, Computational Biology and Complex Systems (BIOCOM-SC) group, Barcelona School of Agri-Food and Biosystems Engineering, Universitat Politècnica de Catalunya, Castelldefels, Spain; ^2^Health Data Sciences, NDORMS, University of Oxford, Oxford, United Kingdom; ^3^Paediatric Infectious Diseases and Immunodeficiencies Unit, Children’s Hospital, Vall d’Hebron Barcelona Hospital Campus, Barcelona, Catalonia, Spain; ^4^Infection and Immunity in Paediatric Patients, Vall d’Hebron Research Institute, Barcelona, Catalonia, Spain

**Keywords:** respiratory infections, epidemic, levels, threshold, effective potential growth, epidemic indicators, bronchiolitis, influenza

## Abstract

**Introduction:**

Bronchiolitis, mostly caused by Respiratory Syncytial Virus (RSV), and influenza among other respiratory infections, lead to seasonal saturation at healthcare centers in temperate areas. There is no gold standard to characterize the stages of epidemics, nor the risk of respiratory infections growing. We aimed to define a set of indicators to assess the risk level of respiratory viral epidemics, based on both incidence and their short-term dynamics, and considering epidemical thresholds.

**Methods:**

We used publicly available data on daily cases of influenza for the whole population and bronchiolitis in children <2 years from the Information System for Infection Surveillance in Catalonia (SIVIC). We included a Moving Epidemic Method (MEM) variation to define epidemic threshold and levels. We pre-processed the data with two different *nowcasting* approaches and performed a 7-day moving average. Weekly incidences (cases per 10^5^ population) were computed and the 5-day growth rate was defined to create the effective potential growth (EPG) indicator. We performed a correlation analysis to define the forecasting ability of this index.

**Results:**

Our adaptation of the MEM method allowed us to define epidemic weekly incidence levels and epidemic thresholds for bronchiolitis and influenza. EPG was able to anticipate daily 7-day cumulative incidence by 4–5 (bronchiolitis) or 6–7 (influenza) days.

**Discussion:**

We developed a semi-empirical risk panel incorporating the EPG index, which effectively anticipates surpassing epidemic thresholds for bronchiolitis and influenza. This panel could serve as a robust surveillance tool, applicable to respiratory infectious diseases characterized by seasonal epidemics, easy to handle for individuals lacking a mathematical background.

## 1 Introduction

Lower respiratory tract infections (LRTIs) are a significant global health burden, causing substantial morbidity and mortality worldwide. According to the Global Burden of Disease (GBD) Study 2019, LRTIs were responsible for approximately 2.5 million deaths and around 500 million infections globally in 2019 (1–3). LRTIs affect individuals of all ages but disproportionately impact children under 5 years of age and older adults. In the former age group, acute LRTIs are a leading cause of morbidity and mortality, with Respiratory Syncytial Virus (RSV) and Influenza Viruses (IVs) being the two most common causes (4).

The RSV causes approximately 70% of bronchiolitis, a seasonal LRTI that is particularly critical in children under 2 years, with 3.5 million hospitalizations and almost 1% of deaths among admitted children, mostly infants <6 months (4). Bronchiolitis is mostly contagious 5 days after infection, and it is associated with respiratory distress, wheezing, apnea, fever, and nasal flaring, although these symptoms are correlated with the severity of the disease and age (5). Similarly, seasonal influenza, caused by IVs, is responsible for a significant burden of LRTIs in children under 5 years, with an estimated 120,000 deaths annually (6). Nonetheless, adults, particularly those with underlying medical conditions, adults over 60 years of age, and pregnant women (7) are the most affected. The World Health Organization (WHO) describes influenza’s clinical manifestations as fever, dry cough, headache, muscle and joint pain, severe malaise, sore throat, and a runny nose (7). Although seemingly mild, according to the Centers for Disease Control and Prevention (CDC), IVs are responsible for an estimated 9–45 million cases, 140,000–810,000 hospitalizations, and 12,000–61,000 deaths annually in the United States (8–10). Moreover, influenza has a shorter incubation period and is mostly contagious between 48 h and 6 days from infection (11).

In Catalonia, a region with about 7.6 million population in Spain, bronchiolitis and influenza are also significant health concerns for patients and healthcare providers (12). Between 10,000 and 15,000 children under two years get infected with RSV seasonally (13), similar to the values that the peak number of total weekly infections of influenza reaches (14). Given the high incidence and substantial morbidity and mortality associated with bronchiolitis and influenza, conducting effective surveillance of these viral infections is crucial. Surveillance can inform public health interventions and guide the allocation of resources to reduce the burden of these infections, including vaccination campaigns, infection control measures, and appropriate clinical management of patients. Besides, it can guide and comfort healthcare providers during the epidemics.

Epidemic indicators are used to guide surveillance in public health domains, some of them are computed empirically and others are estimated from model parameters, such as the well-known reproduction number (R) (15). Due to the nature of this work, mostly empirical indicators will be described, such as incidence, one of the most commonly used. Incidence measures the disease occurrence in a population, and it is often expressed as the number of cases per 100,000 population over a specific period. The CDC also uses cumulative hospitalization incidence and admissions, in addition to deaths, infection fatality ratio and pediatric deaths, to monitor influenza (16–18). Other organizations such as the European Centre for Disease Prevention and Control (ECDC) monitor laboratory data to detect variants of the viruses circulating or compute the percentage of positive tests for respiratory viral infections (RVIs). In addition, they use sentinel groups to estimate the epidemic incidence levels that a disease achieves, in different countries (19). Another useful measure is the growth rate of the epidemic, which is defined as the relative change in cumulated infections from 1 week to the next. Similarly, the empirical reproduction number is used to estimate and monitor the average number of infections that a single individual triggers. Usually computed from mathematical mechanistic models, the empirical reproduction number is a good measure of the stage of an epidemic, and several studies have been made to improve the calculation of this indicator while reducing complexity avoiding complicated models (20–22). This range of indicators helps us to identify potential outbreaks and track the progression of the disease over time, as was evident during the COVID-19 pandemic (23).

In the present study, we aim to define a set of semi-empirical indicators to assess the risk level of seasonal respiratory epidemics, based on both incidence and their dynamics, and considering epidemical thresholds. We base this risk evaluation system on our previously developed method for monitoring COVID-19 (23, 24). By limiting the use of models, we intend to provide a precise surveillance and short-term forecasting tool for healthcare or public health professionals without an expertise in mathematical epidemiological modeling, not to design immediate control plans, but to assist decisions on the relocation of health resources or simply to provide direct knowledge of the current and short-term expected burden.

## 2 Materials and methods

### 2.1 Data collection

We used publicly available data on daily clinical diagnoses of influenza for the whole Catalan population and bronchiolitis in children less than 2 years old, from 1st September 2014 to 31^st^ March 2023. We obtained the data from the Information System for Infection Surveillance in Catalonia (SIVIC) (25) of the Health Department of Catalonia, a database that contains information on clinical diagnoses in Primary Healthcare, usually mostly without microbiological confirmation. However, previous studies showed that clinical diagnoses data are a good proxy of the epidemiological dynamics of respiratory diseases like influenza, because their results have been representative of laboratory confirmed diagnoses and sentinel systems but entail a shorter delay, as demonstrated by Aguilar Martín et al. (26). We used data from children <2 years for assessing bronchiolitis because this is the main age group affected by this disease. Otherwise, influenza is not only focused on a determined age group and can have an impact among the general population.

### 2.2 Data pre-processing

In this study, we divided the data pre-processing into three different stages: two of *nowcasting* and one of smoothing (Supplementary material). The first *nowcasting* approach is to account for the delayed notification or report in medical databases, while the second one is to consider the differences in data reporting (i.e., influenza cases) depending on whether the day of the week is a working day or a holiday. Finally, to the pre-processed data we performed a smoothing 7-day moving average. To facilitate understanding of the two *nowcasting* methods, we provide a more extensive explanation in the following subsections. The aim of this extensive pre-processing is to extract the global dynamics of the diseases by clearing out all the noise present within them. Notwithstanding that, the rest of the study could be implemented simply smoothing the data. Note that all processes and analyses were done using Python and the codes for this paper are available in https://github.com/BIOCOM-SC/cloud-of-codes.

#### 2.2.1 *Nowcasting* delayed reporting or notifications

There is a well-described problem when working with medical diagnostic databases, data are constantly being updated and the true number of infections for a certain day can only be verified after some period of time. However, the general agreement is to use these data after 1 month since being reported, once it has consolidated (27). In this regard, we had been downloading the SIVIC database each week since the beginning of 2021. We performed a week-to-week comparison of the daily reports in the different databases and ascertained that while records were generally coherent after 30 days from their entry, the most recent registers were still being updated. With a retrospective analysis, we intended to define the percentage of data completion for the last 30 entries in the database, and use them to weight the data into a more accurate approximation of the real number of cases.

Since the reporting methods in Catalan healthcare changed substantially during the pandemic, we focused our process only in the datasets downloaded in mid-October, November and December 2022. These datasets were considered consolidated, being more than three months old by the end of the study period. Additionally, their reporting pattern was closer to the ongoing and pre-pandemic ones than that of 2021. We decided to take records after 30 days from entry as ground truths (consolidated data) and iteratively compute the percentage of completion of each day from October to December 2022, ending up with thirty 30-last days iterations. Thereafter, we averaged the results to obtain the mean percentage of completion per each of the days, which we named *reporting weights*:

(1) $$\omega_{j}=\frac{C_{i}}{C_{i+30}},for\;i=1,\ldots ,30\;\mathrm{days},for\;j=day\;1,\ldots ,\mathrm{day}\;30$$

(2) $$\mathrm{reporting}\;\mathrm{weights}=\bar{\omega_{r}}=\frac{\sum_{j=1}^{30}\omega_{j}}{N_{\mathrm{repetitions}}},for\;j=\mathrm{day}\;1,\ldots ,\mathrm{day}\;30$$

In Eq. 1, $\omega_{j}$ states for the normalized percentage of completion, C_i_ is the number of cases reported in a day and C_i + 30_ the number of cases reported for the same day but 30 days later. The *reporting weights* in Eq. (2) ( $\bar{\omega_{r}}$ ) are constructed as the average $\omega_{j}$ for all iterations performed. In our case, N_repetitions_ = 30 since we started computing $\omega_{j}$ from 20/10/2022 to 20/11/2022 and ended at the iteration from 20/11/2022 to 20/12/2022.

Finally, we estimated the daily cases for the last month since the day the data is downloaded from SIVIC as:

(3) $$C_{i}^{\left(i\right)}=\;\frac{C_{i}}{\bar{\omega_{r}}},for\;i=1,\ldots ,30$$

In Eq. (3), $C_{i}^{\left(i\right)}$ states for the estimated diagnoses in the 30 days previous to the last update of the database. Former works share this approximation (24, 28, 29).

To end the pre-processing and smooth the data of bronchiolitis infections, we apply to $C_{i}^{\left(i\right)}$ a cumulative 7-day moving average filter, while the influenza series undergo the weekly pattern correction detailed in the next subsection.

#### 2.2.2 *Nowcasting* weekly patterns

SIVIC data followed a weekly pattern, Mondays having approximately double the cases of weekends or holidays (Supplementary material). However, bronchiolitis cases usually follow a highly stochastic nature thus their pattern of report is not stable nor avoidable. Hence, this approach can only be applied when an evident pattern is present like in the influenza diagnoses.

The main process comprises labeling every day in the study period as Monday (1), Tuesday (2), Wednesday (2), Thursday (2), Friday (2), Saturday (3), Sunday (3) or Holiday (3) as stated by the working calendar in Catalonia for each year. Therefore, we created three groups of days, the regular working days from Tuesday to Friday, the weekends and festivities when the healthcare centers only attend emergencies, and Mondays when all non-urgent cases occurring in weekends are finally attended. In addition, days after a festivity are labeled as Mondays (1) to capture the same effect as described.

Afterwards, we took daily windows of 7-days from the start to the end of the study period and computed the weights per type of day as the difference between the raw number of diagnoses reported in SIVIC and the daily filtered number of diagnoses with a 7-day moving average (MM7), that is:

(4) $$\delta_{j}=\frac{C\;\left(j\right)}{CMM7\;\left(j\right)},for\;j=1,2,3$$

**Figure 1:**
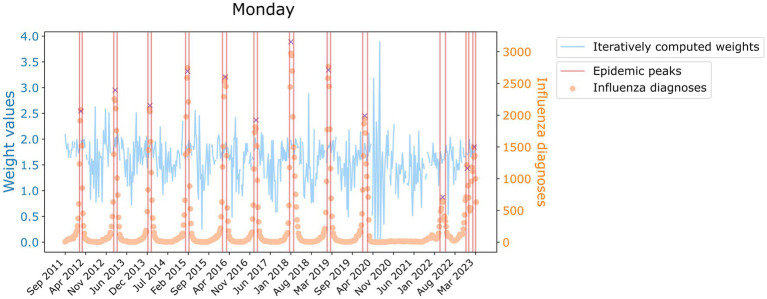
Value of the weight of Monday for its iterative calculations historically (blue, left axis). In orange and referred to the right axis, the time series of influenza diagnoses. Delimited in red, are the zones where the median weight value has been computed. Red crosses indicate the peaks detected.

In Eq. (4), $\delta_{j}$ are the weights computed for Mondays (j = 1), regular workdays (j = 2) and weekends and festivities (j = 3) in a certain 7-days window. C states for the raw daily reports in SIVIC and CMM7 for the 7-day moving mean of C. The j index indicates that to compute a certain weight, we only consider the cases of its kind. We saved all the iterative computations per type of day and graphically observed a time-varying pattern in which stochasticity was reduced when epidemic peaks were reached. Therefore, we took the weights per kind of day as the median among the intervals in which the values were more stable, which were detected with a signal processing algorithm detecting local maxima, as displayed as an example in Figure 1. We decided to take the median value instead of the average to account for instability.

However, we wanted to account for the stochasticity present in the data. Hence, instead of using a constant weight value we used a random Gaussian distribution in which the aforementioned computed weights are the mean of the distribution, but its standard deviation is inversely proportional to the recorded number of diagnoses, ensuring that negative weights are avoided. Consequently, the more daily cases, i.e., the closer to the epidemic peak, the more the final weight resembles the average. Besides, we only apply this modification when CMM7 are over 100 influenza infections, when fluctuations are low enough to have a signal-to-noise ratio large enough to make the computation of weights reliable.

Finally, we applied the weekly reporting pattern to the daily estimated diagnoses $C_{i}^{\left(i\right)}$ that we already computed in order to obtain more balanced data:

(5) $$D=\frac{C_{i}^{\left(i\right)}}{\delta_{j}},for\;i=\mathrm{days}\;\mathrm{in}\;\mathrm{the}\;\mathrm{study}\;\mathrm{period}\;\mathrm{and}\;j=1,2,3$$

In Eq. (5), D represents the final weighted diagnoses. We based this approach on the work of Català et al. (18, 23) and Villanueva et al. (30). Further information can be found in the Supplementary material. To end the pre-processing and smooth the data of influenza infections, we apply to D a cumulative 7-day moving average filter.

### 2.3 Epidemic levels

To define epidemic levels, we employed a novel approach based on the Moving Epidemic Method (MEM) which is used in European institutions such as the ECDC (31, 32). But first, we needed to compute the weekly incidence for each disease and set an epidemic threshold from which to compute these stages.

From the pre-processed dataset, we measured the weekly incidence of bronchiolitis and influenza computing the number of daily diagnoses per 10^5^ population (<2 years and all Catalonia respectively) and resampling them to weekly frequency. To determine the start of the epidemic, we used the first derivative of daily diagnoses. The first derivative represents the rate of change of the number of reported cases with respect to time. By looking at a certain value of the derivative, we can identify the day when the number of reported cases started to increase rapidly. We set this value to a three-fold increase in the number of reported cases over a single day. We then looked for the number of cases reported that day from 2014 to 2019 and averaged them. The exclusion of pandemic years is deliberate to avoid skewing the result. Once we found the epidemic threshold, we selected the epidemy as the first and last days when we are over this boundary.

With the epidemy delimited, we computed an average epidemy among the pre-pandemic ones and calculated the 25th, 50th, 75th and 95th percentiles of cases. The number of cases up to the threshold represent the basal level of the epidemy, from the threshold to the 25th percentile correspond to a very low level of the epidemy, from the 25th to the 50th percentile indicates a low level, from 50th to 75th signifies a medium level, from 75th to 95th represents a high level and above the 95th constitutes very high epidemic levels. Since with this method we obtain epidemic thresholds for weekly incidence, we divide the values obtained by 7 to also have the daily incidence levels. This whole process has been coded in R and is available in (33).

### 2.4 Epidemic indicators

In the present work, we used four different epidemic indicators: the daily incidence of disease, the weekly growth rate, the semi-empirical reproduction number and the Effective Potential Growth (EPG) (24). All of them are computed from the pre-processed datasets.

#### 2.4.1 Daily incidence

To calculate the daily incidence of bronchiolitis and influenza, we took the daily number of diagnoses weighted and filtered with a cumulative 7-day moving average and computed cases per 100,000 population as:

(6) $$I_{i}^{\inf\mathrm{luenza}}=10^{5}.\frac{D_{i}}{P_{i}},for\cdot i=\mathrm{days}\;\mathrm{in}\;\mathrm{the}\;\mathrm{study}\;\mathrm{period}$$

(7) $$I_{i}^{\mathrm{bronchiolitis}}=10^{5}\cdot \frac{C_{i}^{\left(i\right)}}{P_{i}},\cdot for\cdot i=\mathrm{days}\;\mathrm{in}\;\mathrm{the}\;\mathrm{study}\;\mathrm{period}$$

In Eq. (6), $I_{i}^{\mathrm{influenza}}$ states for the daily incidence of the disease, D_i_ represents the pre-processed number of infections in a day and P_i_ the general number of inhabitants of Catalonia. Regarding Eq. (7), $I_{i}^{\mathrm{bronchiolitis}}$ represents the daily incidence of bronchiolitis, $C_{i}^{\left(i\right)}$ the pre-processed number of infections in a day and P_i_ the number of infants <2 years in Catalonia. The population has been considered constant intra-yearly but variable inter-annually.

#### 2.4.2 Weekly growth rate

To assess the weekly growth rate, we first define the weekly incidence as previously explained, resampling daily incidence to weekly frequency. With this, we define the weekly growth rate as the percentage of more (or less) cases reported in a week compared to the previous week:

(8) $$\mu_{j}=\frac{\varphi_{j}}{\varphi_{j-1}},for\;j=\mathrm{all}\;\mathrm{weeks}\;\mathrm{in}\;\mathrm{the}\;\mathrm{study}\;\mathrm{period}$$

In Eq. (8), $\varphi_{j}$ stands for the weekly growth rate, obtained from $\varphi_{j}$ that represents the weekly incidence of disease in a certain week. The higher the weekly growth rate, the faster the disease is spreading.

#### 2.4.3 Effective reproduction number

The effective reproduction number (R) is an estimation of the average number of infections produced by a single infected individual over their infectious period. It is computed taking into account the generation time, which is defined as the average interval between the infection of an individual and the infection of its secondary cases. It usually corresponds to the infectious period. For influenza, the generation time is between γ = 2 and γ =6 days. For bronchiolitis, the generation time is in the order of γ = 5 days. This index is usually computed through the equations of mathematical mechanistic models such as the Susceptible-Infected-Recovered (SIR) model (34). However, it has undergone several redefinitions to enable alternative (rough) estimations without detailed knowledge of specific disease characteristics or the need to solve complex equations (20–22). When the effective reproduction number has temporal resolution, it can be used to predict disease dynamics and evolution. An R > 1 means the number of new infections is increasing while R < 1 indicates that the new infections have decreased over the generation time.

In this work, we define a semi-empirical reproduction number ( $\rho_{\gamma }$ ), as the ratio of new cases with respect to cases γ days ago, with γ the most contagious period of the disease that also corresponds to the time between cases, and filtered with a 3-day moving mean:

(9) $$\rho_{\gamma }^{i}=\frac{D_{i-1}+D_{i}+D_{i+1}}{D_{i-\gamma -1}+D_{i-\gamma }+D_{i-\gamma +1}},for\;i=1,\ldots ,N\;\mathrm{days}$$

In Eq. (9) the semi-empirical reproduction number is presented, with N the number of days of the study period, D the filtered and pre-processed diagnoses either of bronchiolitis (Eq. (3) or of influenza (Eq. (5), and γ=5 both for bronchiolitis and influenza. We decided to use γ=5 for influenza after analyzing the robustness of the results obtained for γ=2 to γ=6 days, which is the interval that literature proposes as time between infections. Since the resulting $\rho_{\gamma }$ , especially for bronchiolitis, were strongly fluctuating, we decided to apply a 7-day moving mean filter to smooth the effects of the stochasticity of certain diagnostic reports.

From all possible estimations of the reproduction number, we decided to use the semi-empirical $\rho_{\gamma }$ , from now on $\rho_{5}$ , (Eq. (9) due to its simplicity. Our aim is not to find the most accurate reproduction number, but an estimate that allows us to make a good monitoring of the dynamics of the epidemic. We intend for professionals without mathematical background to understand epidemic dynamics and indicators, hence so we applied the Occam’s Razor Principle.

#### 2.4.4 Effective potential growth

The Effective Potential Growth (EPG) is based on the one defined for COVID-19 (24). EPG is an epidemic index that combines the incidence level and the incidence trend into a single parameter, and it has shown to be a useful risk indicator for the monitoring of COVID-19. In this work, we defined it as the product of the daily 7-day cumulated incidence of infections (A_7_) by the corrected semi-empirical reproduction number ( $\rho_{\gamma }$ ). Since the time *t* = 7 for A_7_ and the generation interval are different, the reproduction number has to be corrected as:

(10) $$\rho_{\gamma_{c}}={\left(\rho_{\gamma }\right)}^{\frac{t}{\gamma }}$$

(11) $$EPG=A_{7}\cdot \rho_{\gamma_{c}}$$

We defined in Eq. (10) the corrected semi-empirical reproduction number, with *t* = 7 and γ=5 in our particular case. In Eq. (11) we presented the EPG index as the product of A_7_ and the $\rho_{\gamma_{c}}$ , from now on $\rho \prime_{5}$ , afore introduced. The semi-empirical reproduction number is an estimation of how many new infections generates one infected individual. EPG amplifies or narrows the weekly incidence according to whether there has been an increase ( $\rho \prime_{5}>1\Rightarrow EPG>A_{7}$ ) or decrease ( $\rho \prime_{5}<1\Rightarrow EPG<A_{7}$ ) in cases over the last γ days. In this way, the rate of growth is considered when looking at the weekly incidence of infections and we can anticipate a threshold crossing of the epidemic. Hence, the EPG can be interpreted as a *forecaster* of trend changes, the anticipation of which needs to be determined. However, EPG is not a predictor of incidences, but of the dynamic changes in the evolution of an epidemic, anticipating the level of risk to which we are going to be exposed.

The EPG has an advantage over using $\rho_{5}$ or A_7_ alone in that it is more easily interpretable for healthcare or public health professionals. It presents, in incidence terms, the effects of the reproduction number on the evolution of the epidemic. In addition, it can be combined with risk levels to provide a short-term snapshot.

### 2.5 Measure of anticipation

**Figure 2:**
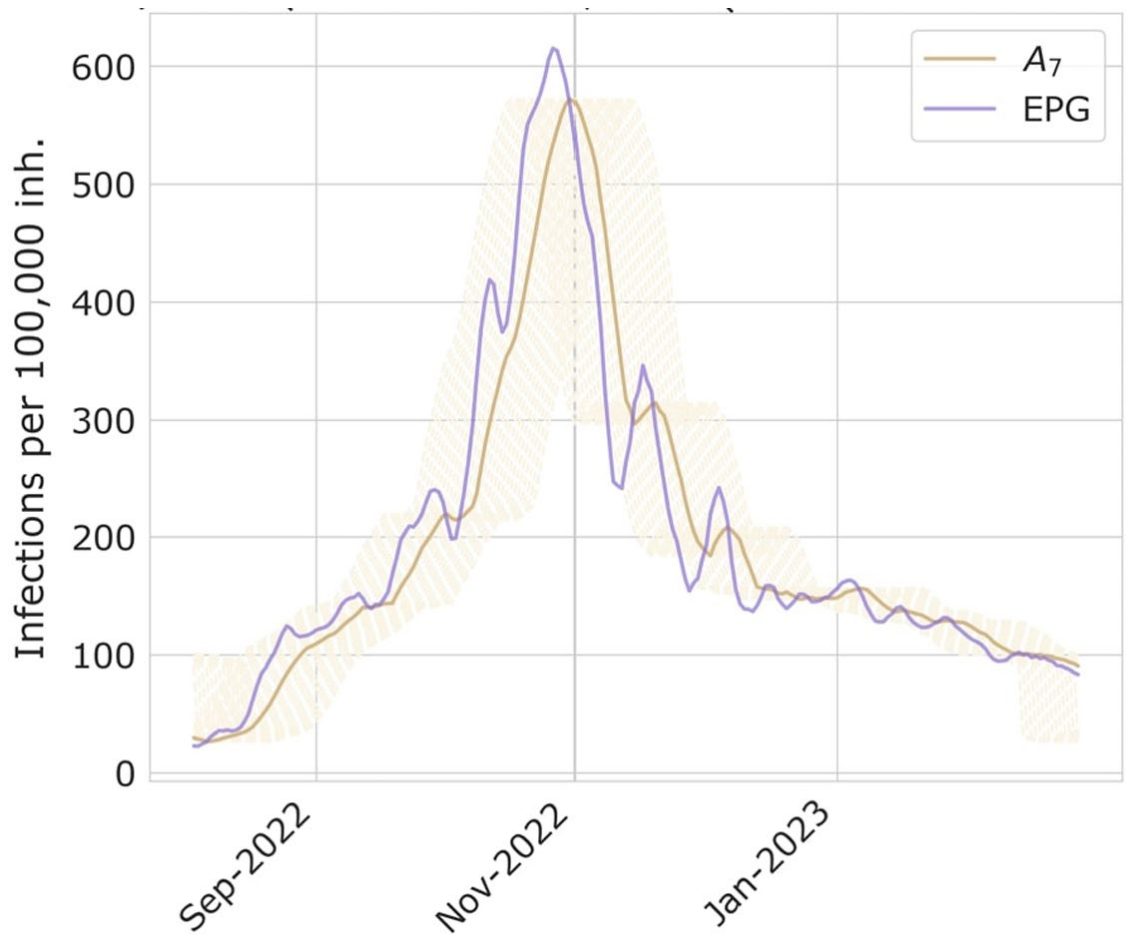
Examples of A_7_ and EPG computed for season 2022–2023 of bronchiolitis, and the process on how to compute the days of delay between both time series peaks.

The objective of creating a monitoring and risk panel for RVIs is not only to assess the current epidemiological situation but to be able to forecast how the course of events will unfold. Subsequently, we performed a Pearson correlation analysis for EPG to determine its suitability and anticipation to the surpassing of epidemic levels. For that, we analyzed how influenza and bronchiolitis incidences correlate and what lag they have with their EPG sequences globally, for their whole series, but also for each of their seasons separately. In Figure 2, you can see a representation of this process, and further visualizations can be found in the Supplementary material. Keeping EPG intact (dark purple line), we move forward or backwards A_7_ (light brown lines) and compute the Pearson correlation among both signals. That way, the anticipation of EPG to A_7_ can be computed, since that number of days will correspond to the strongest correlation coefficient.

We also looked at how many days EPG advances the different epidemic levels, computing the difference in days when a certain threshold is reached.

## 3 Results

### 3.1 Epidemic levels and threshold

**Figure 3:**
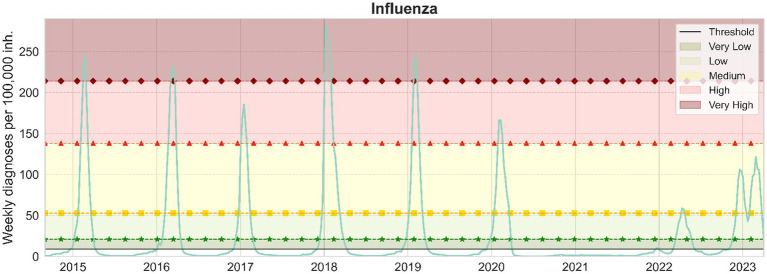
Weekly influenza cases per 100,000 inhabitants in Catalonia. From bottom to top, the epidemic threshold (black), the low (green, stars), medium (yellow, squares), high (red, triangles) and very high (maroon, diamonds) epidemic levels are also displayed.

**Figure 4:**
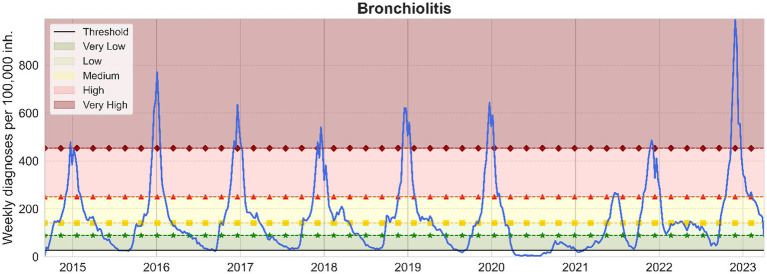
Weekly bronchiolitis cases per 100,000 inhabitants <2 years in Catalonia. From bottom to top, the epidemic threshold (black), the low (green, stars), medium (yellow, squares), high (red, triangles) and very high (maroon, diamonds) epidemic levels are also displayed.

After performing the extensive pre-processing, we obtain smooth visualizations of daily number of diagnoses of bronchiolitis and influenza throughout the study period. With them, we have been able to compute daily and weekly incidence of disease, allowing us to define epidemic stages. The resulting computations of daily and weekly epidemic incidence threshold and levels for influenza and bronchiolitis are collected in Table 1. Furthermore, weekly thresholds are represented in Figures 3, 4, for influenza and bronchiolitis respectively, together with their weekly incidences.

**Table 1:** Epidemic threshold and levels of daily and weekly incidence for influenza and bronchiolitis diseases.

Level	Daily		Weekly	
	Influenza (diagnoses/10^5^)	Bronchiolitis (diagnoses/10^5^)	Influenza (diagnoses/10^5^)	Bronchiolitis (diagnoses/10^5^)
Threshold	1	4	9	27
Low	3	13	21	89
Medium	8	20	53	141
High	20	36	138	250
Very high	31	65	214	453

These results suggest that when we have a weekly incidence of 9/27 or a daily incidence of 1/4 for influenza/bronchiolitis, we can consider the epidemic wave to have started and we will remain at very low numbers of infections until we cross the low epidemic thresholds, after which we should already observe effects at the level of occupancy in health care facilities.

We can notice from Figures 3, 4 the disappearance of influenza when the COVID-19 pandemic spread in March 2020, until mid-2022 when a small epidemic occurred. Meanwhile, diagnoses of bronchiolitis were reported in winter 2020 and two consecutive relatively small epidemics in 2021, both during summer and winter, surpassing the epidemic thresholds defined but not reaching very high levels.

Nonetheless, as Figure 4 shows, the latter epidemic of bronchiolitis has been the historically greatest appearing 1 month earlier. As concerning influenza, in Figure 3 we can ascertain that we are still moving toward a new “normal” seasonality. The latter epidemic wave of influenza was advanced also 1 month from previous seasons, and actually consisted of two different outbreaks, the first one mainly corresponding to influenza A and the subsequent to mainly influenza B viruses (35).

On another note, these visualizations allow us to contrast the nature of both diseases. Bronchiolitis is of a highly stochastic nature, partially because it affects a smaller population (only children) and because the disease can be caused by several viral agents creating plateaus before and after the epidemic peak, which is mostly caused by RSV. On the other hand, influenza presents a smoother signal, both because the number of daily diagnoses is higher and because in Catalonia only two different strains of influenza viruses, A and B, are widespread (32).

Comparing Figures 3, 4, the distance between the low and medium epidemic thresholds is narrower for bronchiolitis than for influenza, as an effect of that previously described *plateau* present in the bronchiolitis infections data. This indicates that for bronchiolitis, the epidemic thresholds defined might only be useful from the medium threshold, when the clear epidemic wave started before the pandemic. Besides, we still have to be cautious with the levels calculated since there are still many unknowns about how future epidemics of influenza and bronchiolitis will unfold in Catalonia after COVID-19.

### 3.2 Effective potential growth

**Figure 5:**
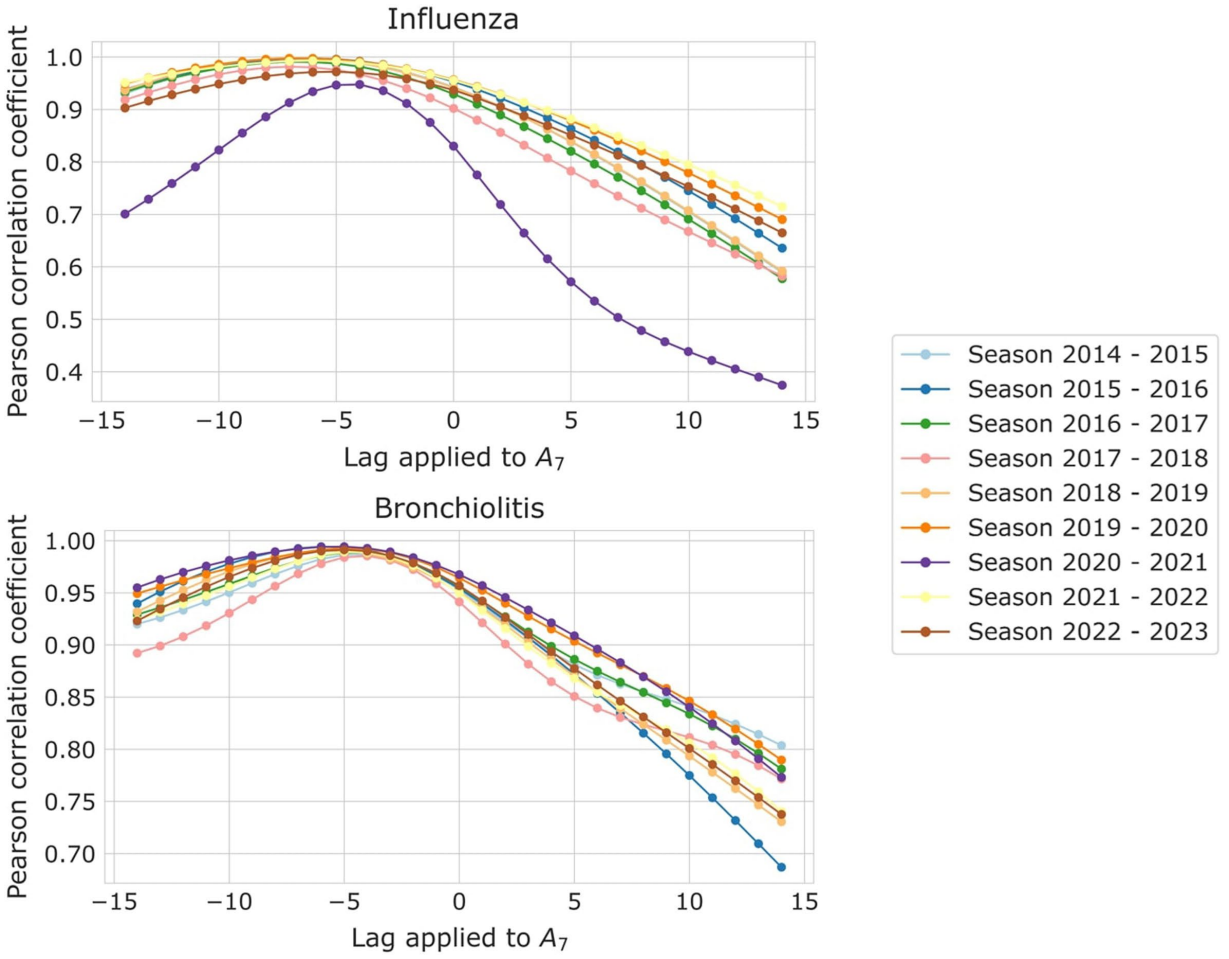
Pearson correlation coefficient among A_7_ and EPG, for the different lags applied to A_7_, for (top) influenza and (bottom) bronchiolitis, and the different epidemic seasons, each corresponding to a different color.

With a correlation coefficient higher than 0.98, we found that the EPG anticipates weekly influenza incidence by 6 to 7 days and bronchiolitis by 4 to 5 days. In Figure 5 we provide the results of the correlation analyses for both diseases.

It is noteworthy that the EPG effectively predicted the incidence of bronchiolitis and influenza almost a week in advance, maintaining strong correlation coefficients even during and after the pandemic. During the first epidemic following the SARS-CoV-2 outbreak, it experienced only a slight decrease in predictive ability, losing 1–2 days of anticipation. However, the 2020–2021 epidemic period should be excluded from the analysis due to the negligible incidence of influenza and the low occurrence of bronchiolitis cases.

We also looked at how many days EPG anticipates the different epidemic levels, and the results are collected in Table 2.

**Table 2:** Number of days in which EPG advances the reaching of the different epidemic thresholds with respect to A_7_.

Bronchiolitis				
Season	Threshold			
	Low	Medium	High	Very high
2014–2015	>10	9	6	6
2015–2016	7	>10	7	10
2016–2017	7	>10	7	9
2017–2018	5	9	6	8
2018–2019	6	9	>10	6
2019–2020	>10	>10	7	>10
2020–2021	9	-	-	-
2021–2022	0	5	>10	>12
2022–2023	0	6	>10	4

Influenza				
Season	Threshold			
	Low	Medium	High	Very high
2014–2015	8	3	8	8
2015–2016	>10	>10	10	7
2016–2017	7	6	7	–
2017–2018	5	5	6	8
2018–2019	>10	8	6	9
2019–2020	>10	6	7	–
2020–2021	–	–	–	–
2021–2022	9	5	–	–
2022–2023	0	>10	–	–

For (top) bronchiolitis and (bottom) influenza diseases.

Notice that not all columns in Table 2 are filled. That is because not all epidemic seasons reach all the different thresholds, some of them only achieve medium levels of incidence. In addition, the robustness of EPG in influenza anticipation is palpable when compared to the results for bronchiolitis, a consequence of the nature of the data used, with much less daily diagnoses than influenza. Hence the bronchiolitis reports present and therefore can cause artifacts leading to less robust results, presented as >10 days. For the same reason, for bronchiolitis, only EPG anticipating high and very high risks should be considered, since lower incidences still present reporting variability that adds noise to the metric. Medium risk is also faithfully anticipated, but one should be cautious as to read the results because artifacts appear in some seasons as a result of the plateaus occupying these incidence ranges, plateaus caused by the many viruses that can produce bronchiolitis before the RSV predominates.

**Figure 6:**
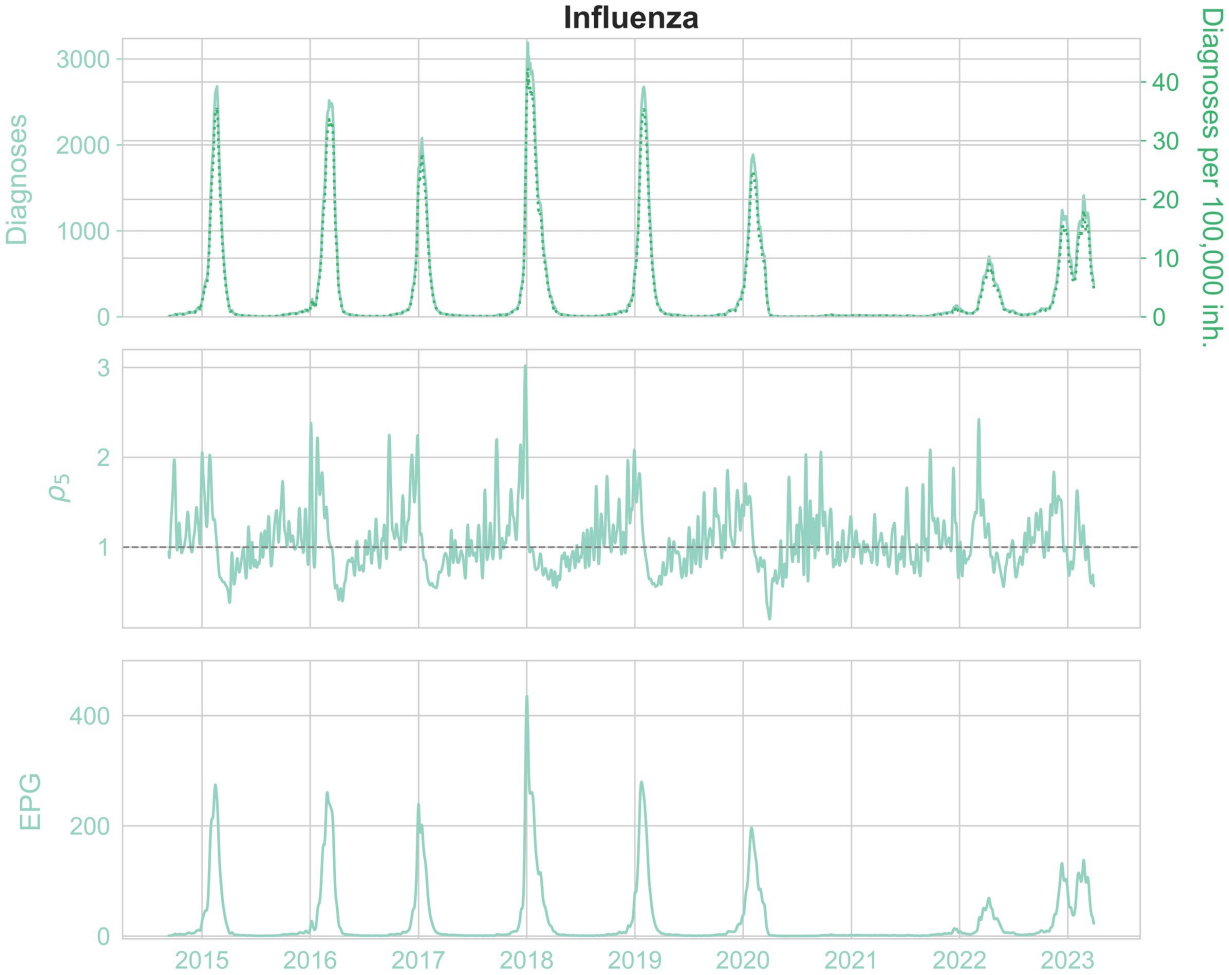
From top to bottom, the daily diagnoses (left) and daily diagnoses per 100,000 population (right, pointed), ρ_5_ rate and EPG (weekly) infections per 100,000 population, for influenza.

**Figure 7:**
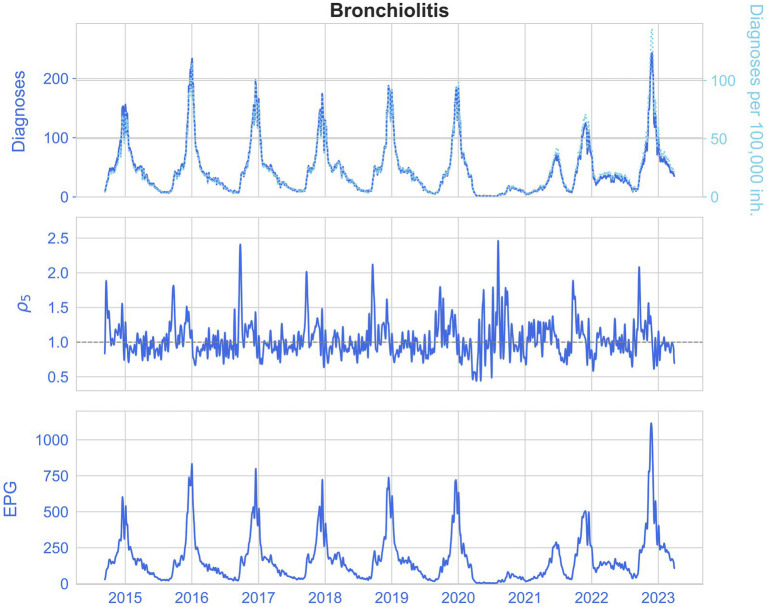
From top to bottom, the daily diagnoses (left) and daily diagnoses per 100,000 population (right, pointed), ρ_5_ rate and EPG (weekly) infections per 100,000 population, for bronchiolitis.

For further insight into the results, we present the historical diagnoses, incidences, ρ_5_ and EPG measurements in Figures 6, 7 for influenza and bronchiolitis, respectively.

Once again, the stochastic nature of epidemic medical records is ascertained, in particular when looking at the estimated reproduction numbers ρ_5_. In addition, we see how before an epidemic peak there is a raise of ρ_5_ up to 3, which means that a large number of contagions are taking place.

The similarity between the incidence of diagnoses and the EPG for both diseases can be corroborated, as well as the slight advancement of EPG, and how it reaches incidences higher than the equivalent weekly diagnoses, due to prompt growths in infections. This way, it indicates the risk of growth of an epidemic.

### 3.3 Risk diagrams

**Figure 8:**
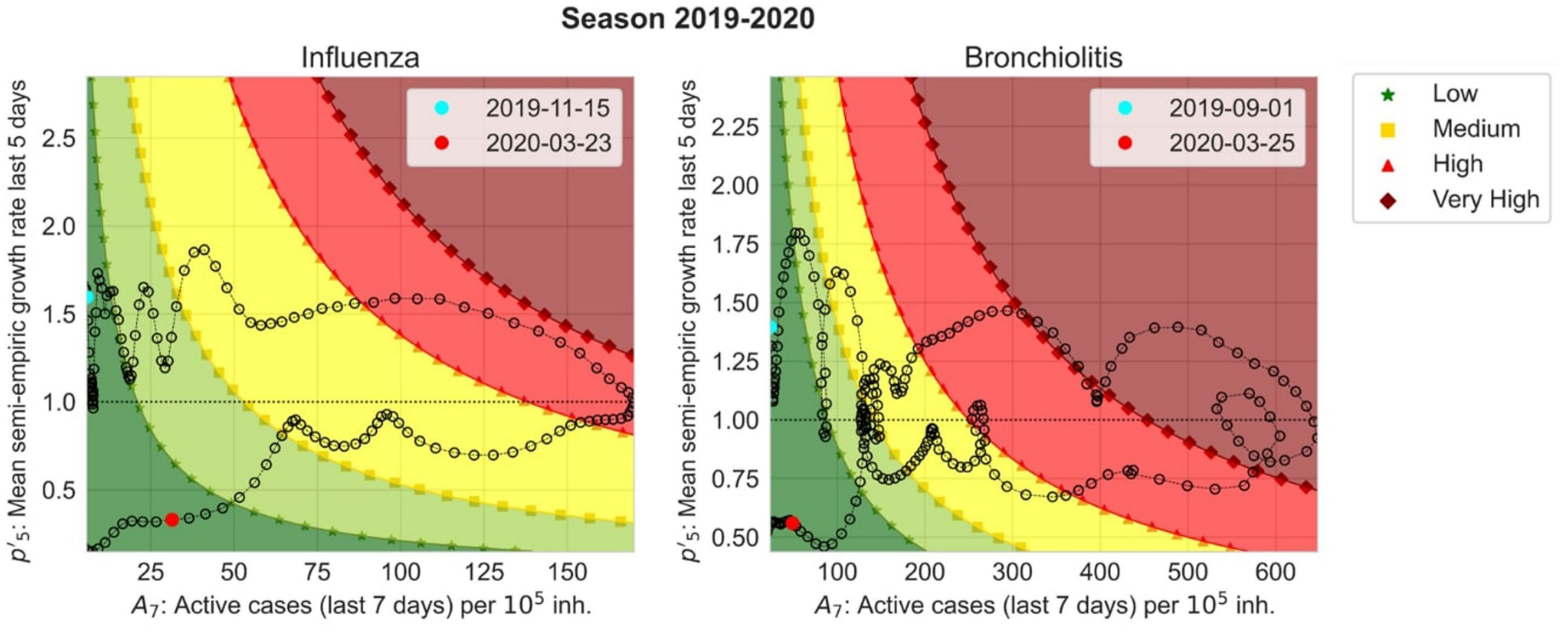
Risk diagrams for season 2019–2020 for influenza (left) and bronchiolitis (right). They show $\rho \prime_{5}$ with respect to A_7_ starting from the cyan point and finishing at the red point. The background colors correspond to EPG values classified by the epidemic levels. Very low (dark green) and low (light green) levels are separated by “*”, low and medium (yellow) levels by a square, medium and high (red) levels by triangles and high and very high (maroon) levels by diamonds.

To assist the visualization of EPG to better interpret it, we plotted the so-called risk diagrams (24) in which we have $\rho \prime_{5}$ in front of A_7_ and a shaded background in a color scale representing the different epidemiological levels defined: dark green for very low or basal level, light green for low, yellow for medium, red for high and maroon for very high weekly incidence levels. To enhance readability and assist all readers, we have incorporated distinct symbols in our presentation. We differentiate between very low and low levels by “*,” low and medium levels by a square, medium and high levels by triangles and high and very high levels by diamonds. The growth/decrease threshold ( $\rho \prime_{5}$ = 1) is shown as a dotted line. Each dot in the plot depicts an EPG value for the corresponding A_7_ and ρ_5_ in a certain day, and the dashed line joins two consecutive days. The more separated the points, the greater the increase or decrease in incidence (horizontal direction) or growth rate (vertical direction). The day the epidemic threshold is crossed initially is drawn as a blue dot and the final day of the epidemy, when we cross that value again, is in red. The x-axes are limited to only show A_7_ incidences above the weekly epidemic threshold. An example of risk diagram can be found in Figure 8 but the complete set of risk diagrams for all epidemic seasons during the period of study can be found in the Supplementary material.

The risk diagrams allow us to anticipate the evolution of an epidemic in a very straightforward way. If we have an influenza incidence of 50 cases per 10^5^ inhabitants but we are above the dotted line that separates growth from decrease, we expect that the number of active cases will continue to increase, following the pattern of the last 5 years. On the other hand, if the same incidence is located below the dotted line, it will not. Then, the color scale helps us to define where we are in the epidemic, whether we are at low (dark and light green), medium (yellow) or high (bright and dark red) incidence values.

### 3.4 Surveillance table

**Figure 9:**
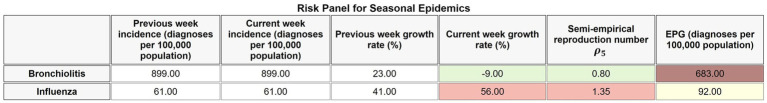
Capture of the risk panel for seasonal epidemics in Catalonia, as of 5th of December 2022.

To enhance and simplify surveillance of respiratory diseases in Catalonia, and facilitate the visualization of the epidemiological indicators, we have developed an automatized control panel, as depicted in Figure 9, that displays the weekly incidence rates for the previous and current weeks, the growth rates for the previous and current weeks, and the EPG. These data are updated daily and the weekly incidence rates and growth rates are calculated by grouping the reported diagnoses over the last 7 days. In Figure 9 we represented the panel at 5^th^ December 2022, when the epidemic of bronchiolitis started vanishing and the influenza wave started to increase.

The last three columns present a color scale such that *Current week growth rate (%)* is green if it is lower than the previous week one, orange if it is the same and red if it is higher; *Semi-*e*mpirical reproduction number* $\rho_{5}$ is green when below 1, orange if equal to 1, and red if greater than 1; and *EPG (diagnoses per 100,000 population)* is white if the epidemic threshold is not surpassed, dark green if we are in very low level, green for low level, yellow for medium level, orange for high level and maroon for very high level. In Figure 9, we observe that we are in a period where the bronchiolitis epidemic is over and we are slowly decreasing incidence, although maintained in high incidences, while at the height of the flu epidemic, with a high number of infections and moving toward greater incidences.

## 4 Discussion

We redefine an epidemic indicator named Effective Potential Growth (EPG) that potentially anticipates changes in weekly epidemic incidence by 4 to 5 days for bronchiolitis and 6 to 7 days for influenza disease. This index, together with a semi-empirical reproduction number, the weekly changes in incidences and growth rates, are the core of the epidemic surveillance panel we developed based on previous work that showed how this approach could work for COVID-19 monitoring (23).

Since the SARS-CoV-2 pandemic, the need for epidemiological surveillance of infectious diseases, especially respiratory diseases, became evident, as airborne transmission is highly effective (36). Several countries already have established publicly available epidemic surveillance systems and outbreak risk indicators, such as the USA (37), UK (38), Canada (39), Australia (40), and even Spain, in particular Catalonia (25). Nonetheless, we have not found any that anticipate the evolution of an epidemic wave targeting a general public without a strong mathematical background. Mathematical modeling of infectious diseases is highly dependent on the quality of the epidemiological data available, and requires expertise to produce and understand the models’ results and parameters. Even though being the most accurate way of forecasting infectious diseases, models can be counter intuitive for medical practitioners or policy makers, who may require of previous formation in the topic. That is why EPG can be a support index for very short-term forecasting, since it shows in advance when the different epidemic thresholds will be achieved and can anticipate by almost a week the epidemic peak. This information is given in incidence terms, which is a common measure of the state of a disease in both public health policy and in medical settings. In addition, we introduce the visualization of EPG in a risk diagram, which can give an illustration of the state of the epidemic in a very straightforward manner. Besides, this epidemiological indicator could be introduced in mathematical models to enhance their prediction capability.

We have also shown that, with a proper pre-processing of the data, and taking into account the weekly differences in reporting, the epidemic waves of influenza and bronchiolitis have had clearly defined thresholds in the last decade, at least in the Catalan healthcare system. These thresholds are particularly robust to different analyses that deal with data reporting fluctuations and, even in the case of bronchiolitis data, where more artifacts are present, they provide an accurate picture of its short-term evolution. Indeed, when the number of weekly cases reaches a certain threshold with a certain weekly growth, a large wave of cases appears in subsequent weeks systematically. For future surveillance, this can be a crucial input to warn the health care system a few weeks in advance of the increase in workload.

Certainly, our proposed scheme has some limitations and the EPG indicator is more robust for influenza than for bronchiolitis, in particular until the medium level threshold. That is due to the stochastic nature of bronchiolitis data, as stated before, and because of the *plateau* present in its epidemic waves. Nonetheless, in most cases we are able to anticipate the change in epidemic threshold by approximately a week in advance. Another limitation is the simplicity of the calculation of the effective reproduction number, which might not be accurately describing the epidemic dynamics. However, is within the error that we accept in exchange for simplicity of interpretation, and we observe that it performs adequately. We could also use it in an anticipatory way, but this is not the objective of this work, since its output is more complex to interpret than that of the incidence, which is why we rely on the EPG.

In addition, currently, hospitalizations are not publicly available, which restricts us to using only primary healthcare data. With hospital admissions, further information could be introduced in our risk panel, such as the severity of the infections by a certain disease, including the ratio of people admitted to the hospital versus clinical diagnoses in primary healthcare, or the percentage of Intensive Care Units (ICUs) occupied. From these data, other risk indicators could be designed, such as an ICU-increase associated risk indicator. Besides, data on mortality could also be a good indicator of the sternness of the disease, but these data are not provided in a daily manner in our region. Additionally, preprocessing medical records is a hard task and there is not a standardized way to do so, yet. The bronchiolitis diagnoses’ stochasticity limits both our preprocessing and predictions abilities with the disease.

Nevertheless, the availability of centralized databases with primary care clinical diagnoses has been enhanced by the pandemic, thus providing a rapid way to monitor the evolution of an epidemic. The panel of semi-empirical indicators that we have presented can be easily incorporated to such databases due to their empirical nature, thus becoming a simple and useful tool to help on the management and surveillance of such epidemic episodes. Our proposed preprocessing methodology allowed us to work with smoother and more reliable data and the defined monitoring panel is the only one to our knowledge using mostly empirical data to construct forecasting indicators, with concept and visualization easy to understand for healthcare practitioners and the general public.

## Data availability statement

The original contributions presented in the study are included in the article/Supplementary material, further inquiries can be directed to the corresponding author.

## Author contributions

AP-M: Conceptualization, Data curation, Formal analysis, Investigation, Methodology, Software, Validation, Visualization, Writing – original draft. MB: Methodology, Software, Validation, Writing – review & editing. VL: Formal analysis, Investigation, Methodology, Supervision, Validation, Writing – review & editing. MC: Conceptualization, Methodology, Supervision, Validation, Writing – review & editing. SA: Conceptualization, Formal analysis, Funding acquisition, Methodology, Supervision, Validation, Writing – review & editing. EÁ-L: Conceptualization, Formal analysis, Methodology, Supervision, Validation, Writing – review & editing. DL: Conceptualization, Methodology, Supervision, Validation, Writing – review & editing. AS-A: Conceptualization, Formal analysis, Funding acquisition, Investigation, Project administration, Resources, Supervision, Validation, Writing – original draft, Writing – review & editing. CP: Conceptualization, Formal analysis, Funding acquisition, Investigation, Methodology, Project administration, Resources, Supervision, Validation, Writing – original draft, Writing – review & editing.
