# Supplementary material for: A semi-empirical risk panel to monitor epidemics: multi-faceted tool to assist healthcare and public health professionals
Source: Front Public Health. 2024 Jan 8;11:1307425. doi: 10.3389/fpubh.2023.1307425 (PMC10801172; doi:10.3389/fpubh.2023.1307425)
Supplement: Supplementary file 1 [file Data_Sheet_1.PDF]

Supplementary Material

1 Pre-processing flowchart

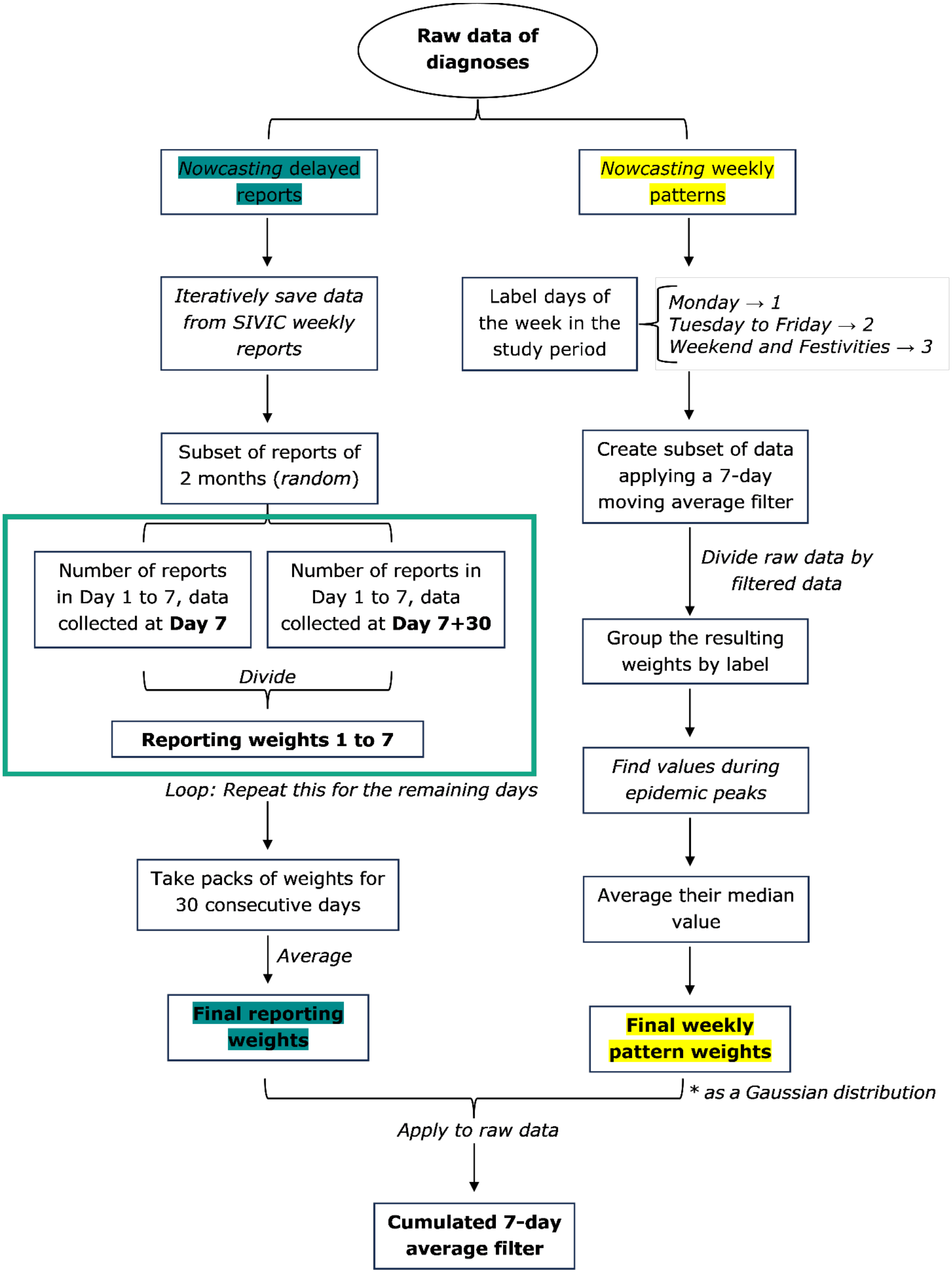

2 Reporting pattern

Supplementary Figures 1 and 2 present the raw data of bronchiolitis and influenza diagnoses, respectively, both historically and zoomed in seasons 2016-2017 and 2017-2018, for further insight. As one can see, the reporting pattern is very straightforward for influenza but is not as regular for bronchiolitis. Hence, only the influenza pattern has been assessed.

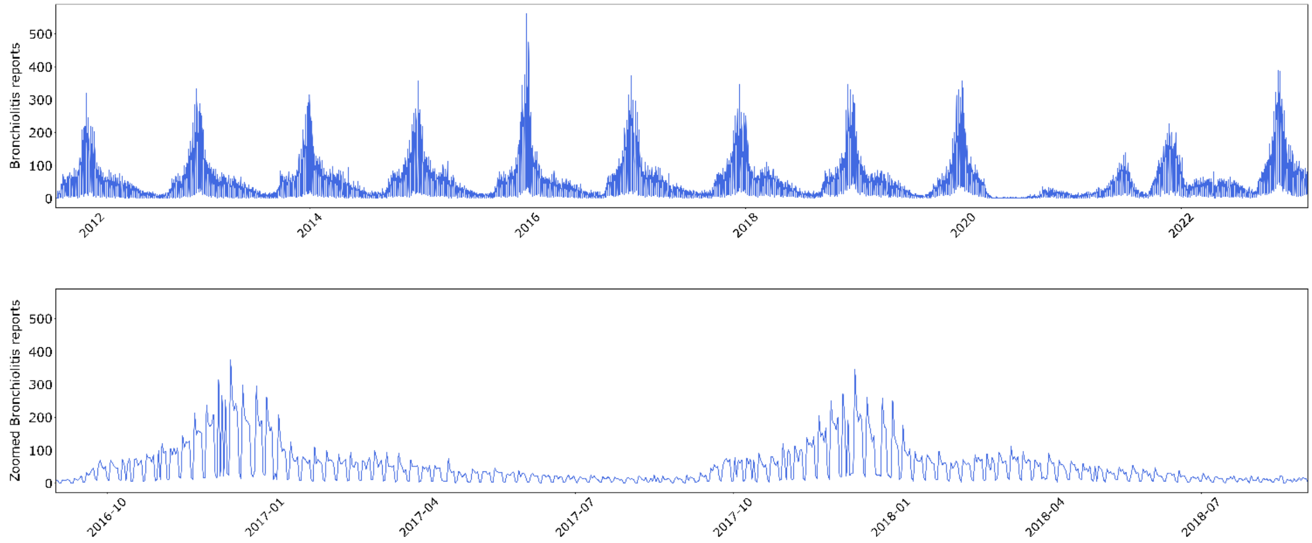

Supplementary Figure 1. Bronchiolitis reports in the SIVIC database historically (top) and zoomed for seasons 2016-2017 and 2017-2018.

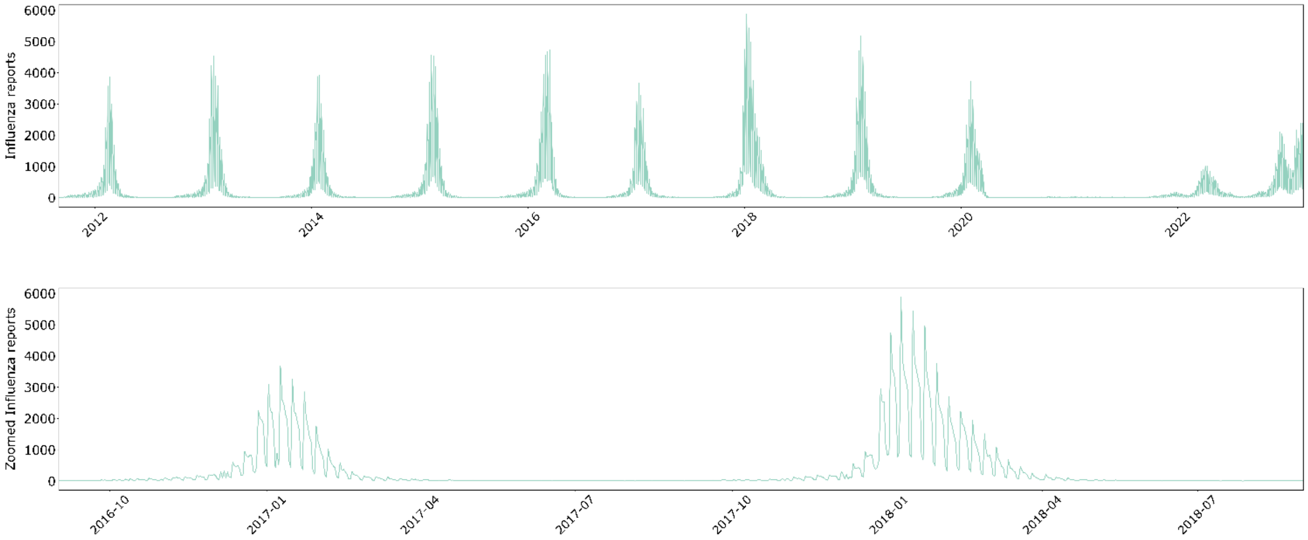

Supplementary Figure 2. Influenza reports in the SIVIC database historically (top) and zoomed for seasons 2016-2017 and 2017-2018.

2.1 Reporting pattern weights

For influenza, we applied a pre-processing in which the weekly pattern of reports in Catalan healthcare centers is considered. In **Supplementary Figure 3**, one can see the reporting weights δ_j obtained.

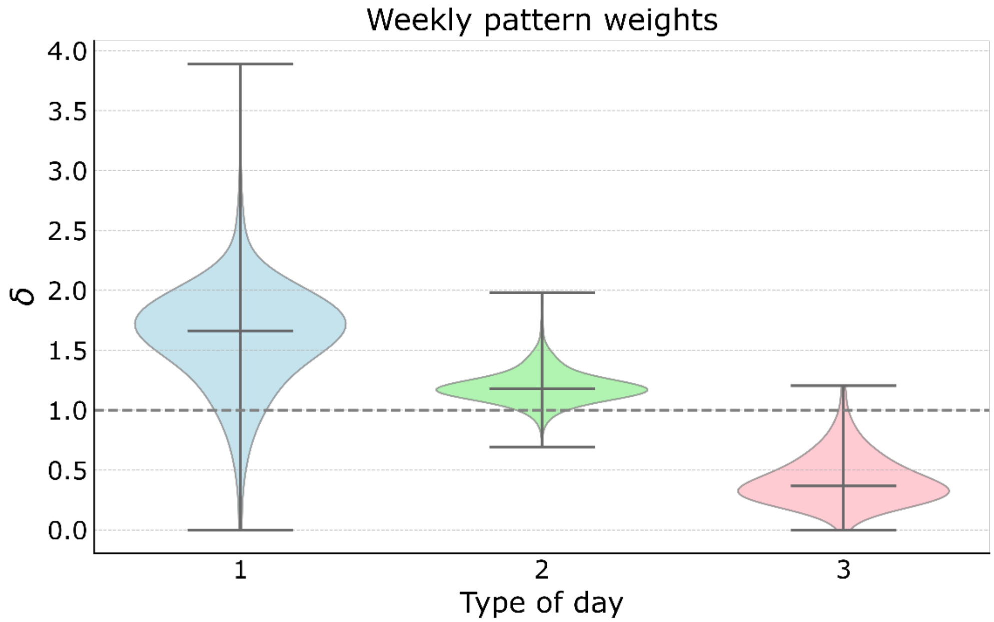

Supplementary Figure 3. Distribution of the values of the weights iteratively computed per each type of day (1) Mondays, (2) workdays and (3) weekends and festivities, for the whole study period.

Due to the high variability in the weights, we only considered their values when an epidemic peak was occurring, since there was less stochasticity in the values for that period, as shown in **Supplementary Figure 4**. Taking into account the weights within red vertical lines, we calculated their median value and take that as the weight per type of day.

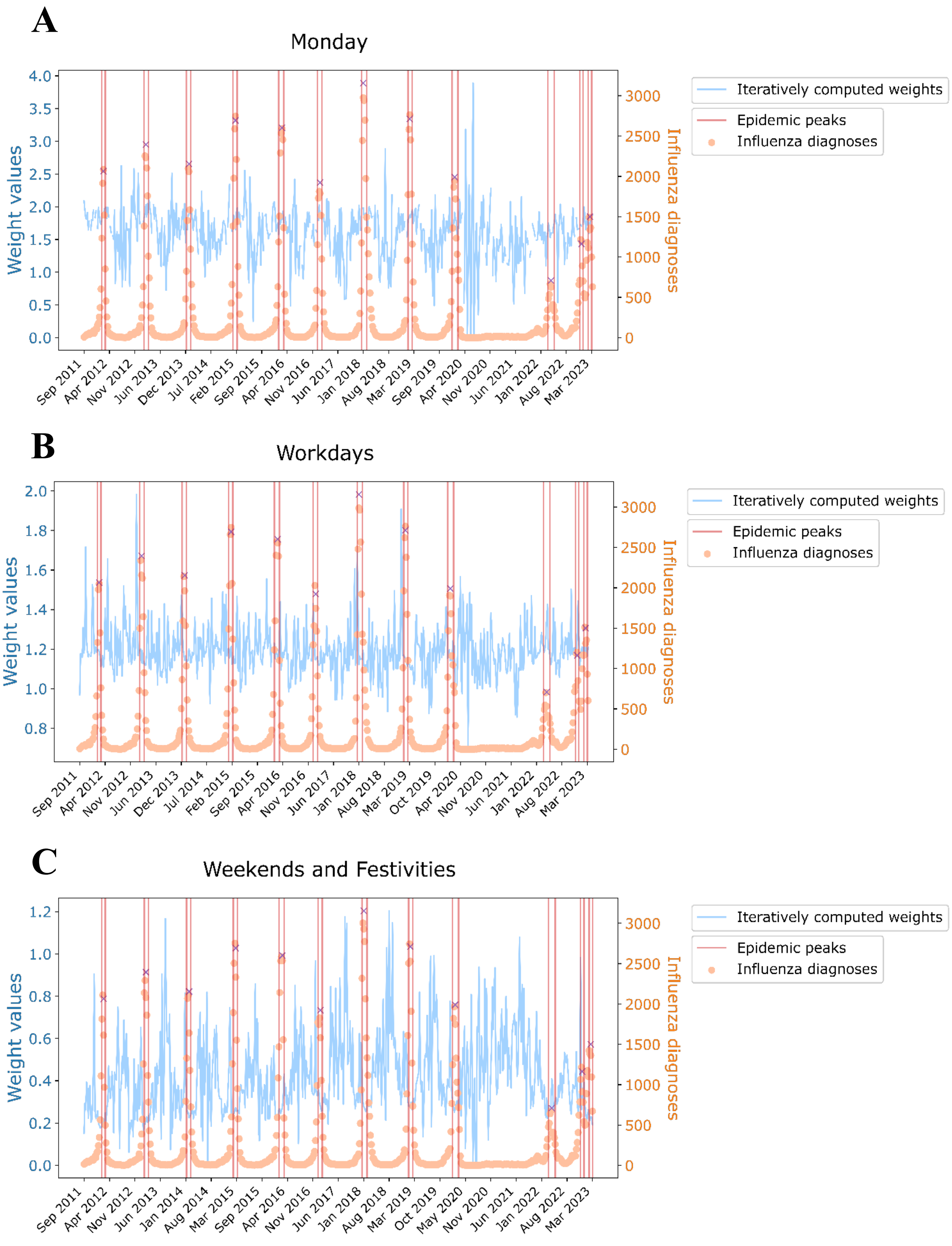

Supplementary Figure 4. Value of the weight of (A) Monday, (B) workdays, (C) weekends and festivities, for their iterative calculations historically (blue, left axis). The time series of influenza diagnoses are in orange and referred to the right axis. Delimited in red, are the zones where the median weight value has been computed. Red crosses indicate the peaks detected.

3 Epidemic levels and threshold

We also computed epidemic threshold and levels from the pre-processed weekly incidence data of bronchiolitis and influenza. For that, we calculated the mean of the pre-pandemic seasons, and from that averaged season obtained the day where the derivative surpassed 3, i.e., the cases were three-folded, and the 25th, 50th, 75th and 95th percentiles from that limit. **Supplementary Figures 5 and 6 A** show the process for influenza and bronchiolitis, respectively. In **Supplementary Figures 5 and 6 B**, on the other hand, the obtained epidemic levels are represented on top of each season.

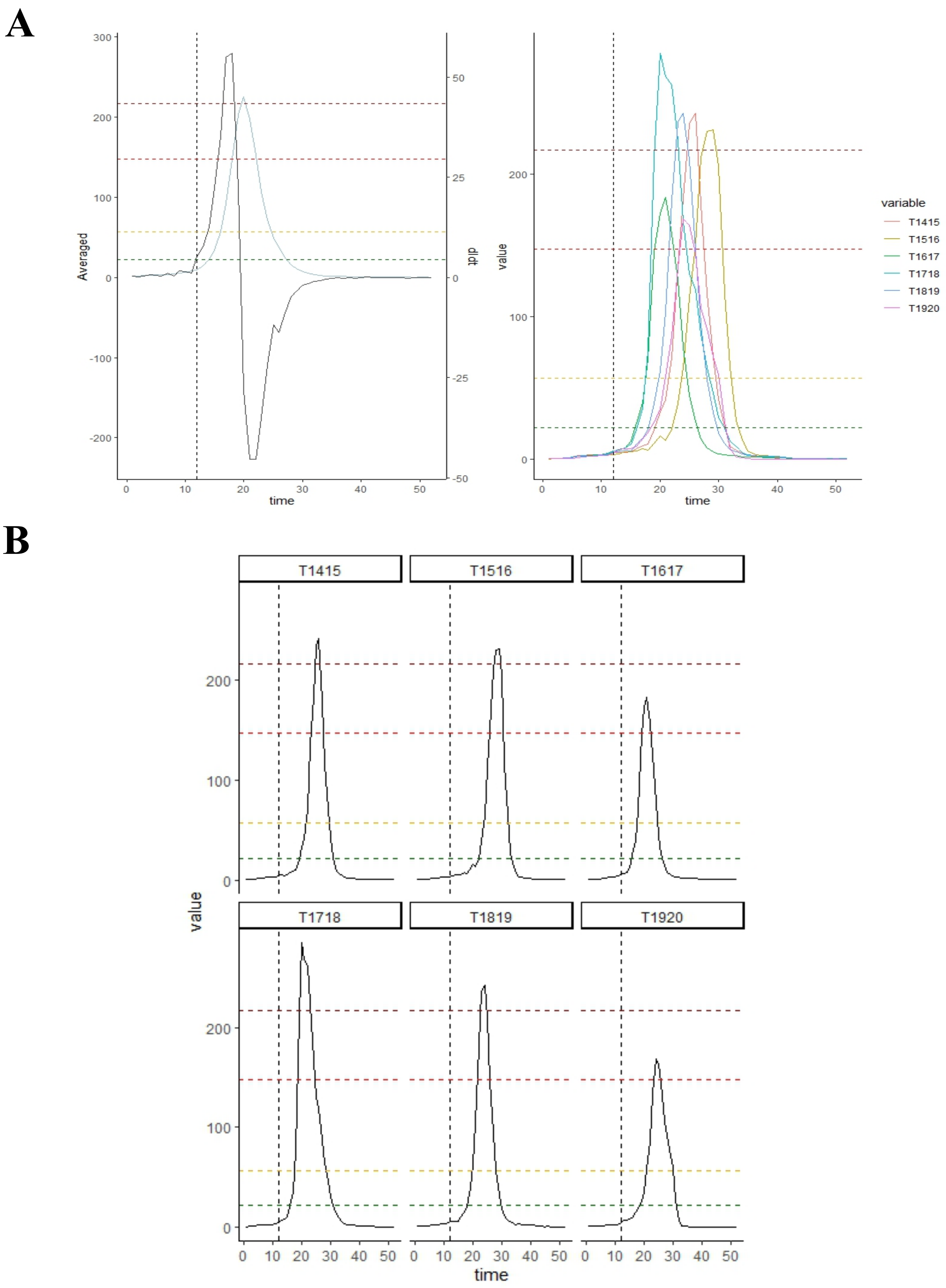

Supplementary Figure 5. In (A, left) the averaged pre-pandemic season of influenza, in (A, right) all pre-pandemic seasons plotted together, and in (B) each of the pre-pandemic seasons individually. With the threshold value in a black vertical dashed line and the levels computed in horizontal dashed lines, green for low, yellow for medium, red for high and maroon for very high, level thresholds.

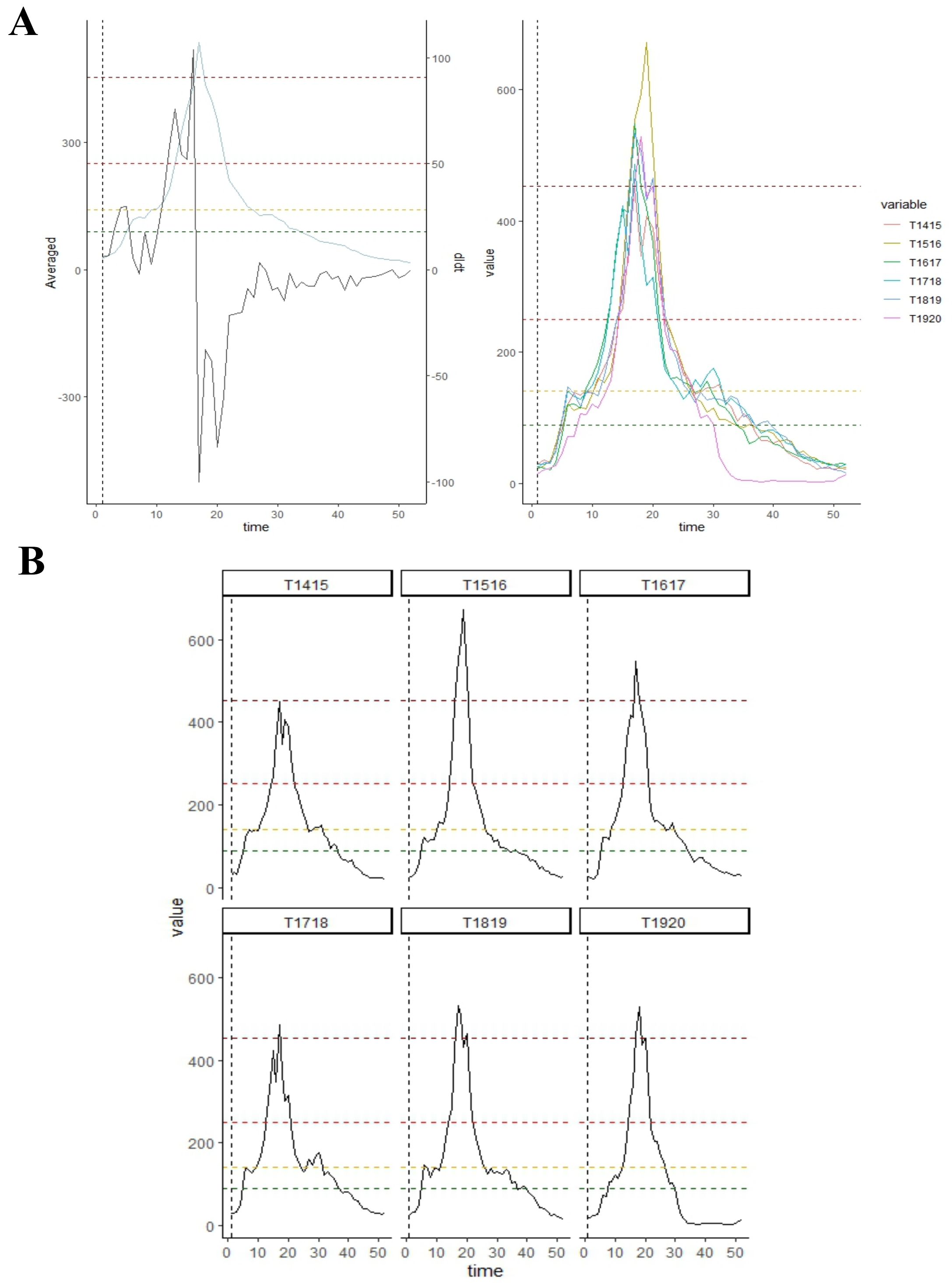

Supplementary Figure 6. In (A, left) the averaged pre-pandemic season of bronchiolitis, in (A, right) all pre-pandemic seasons plotted together, and in (B) each of the pre-pandemic seasons individually. With the threshold value in a black vertical dashed line and the levels computed in horizontal dashed lines, green for low, yellow for medium, red for high and maroon for very high, level thresholds.

4 Effective Potential Growth: Risk Diagrams

On another note, we computed the Effective Potential Growth indicator, that is the product of A_7 and ρ_γ, the 7-day cumulated incidence and the estimated reproductive number, respectively. We represent them in the so-called risk diagrams for the different seasons, which are **Supplementary Figures 7-15**. The shaded background in a color scale represents the different epidemiological levels defined: soft green for very low or basal level, green for low, yellow for medium, orange for high and red for very high weekly incidence levels. The growth/decrease threshold (ρ_γ=1) is shown as a dotted line. Each dot in the plot depicts an EPG value for the corresponding A_7 and ρ_γ in a certain day, and the dashed line joins two consecutive days.

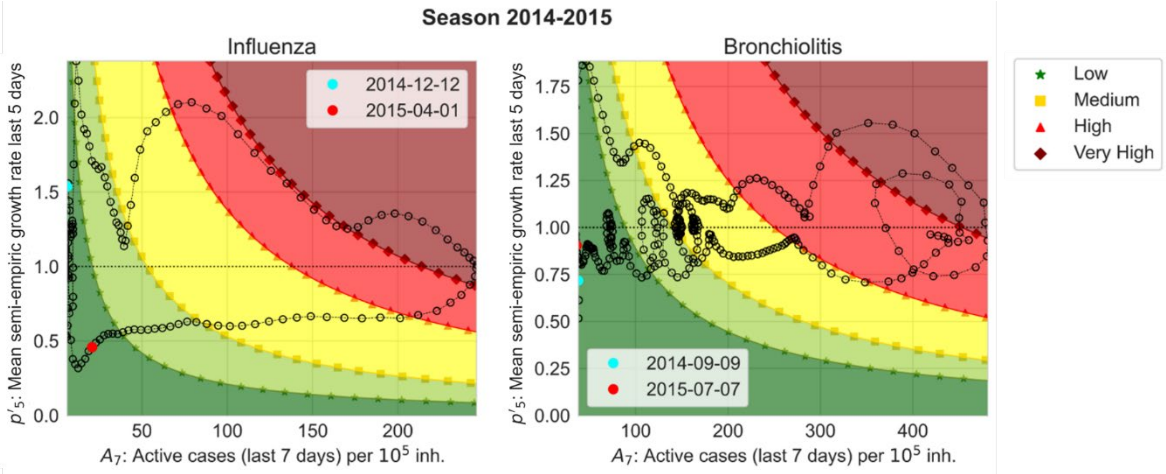

Supplementary Figure 7. Risk diagrams for season 2014-2015 for influenza (left) and bronchiolitis (right). They show ρ'_5 with respect to A_7 starting from the cyan point and finishing at the red point. The background colors correspond to EPG values classified by the epidemic levels. Very low (dark green) and low (light green) levels are separated by "*", low and medium (yellow) levels by a square, medium and high (red) levels by triangles and high and very high (maroon) levels by diamonds.

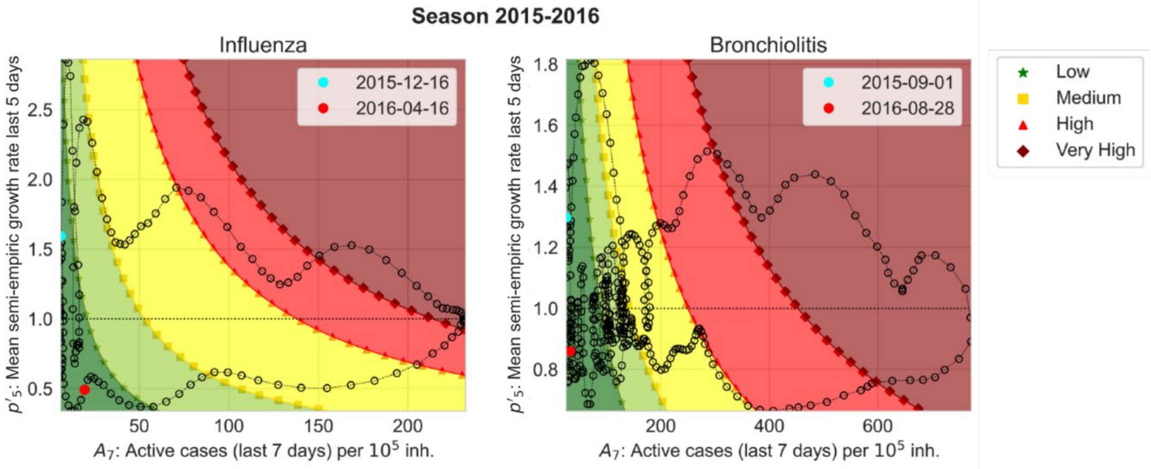

Supplementary Figure 8. Risk diagrams for season 2015-2016 for influenza (left) and bronchiolitis (right). They show ρ'_5 with respect to A_7 starting from the cyan point and finishing at the red point. The background colors correspond to EPG values classified by the epidemic levels. Very low (dark green) and low (light green) levels are separated by "*", low and medium (yellow) levels by a square, medium and high (red) levels by triangles and high and very high (maroon) levels by diamonds.

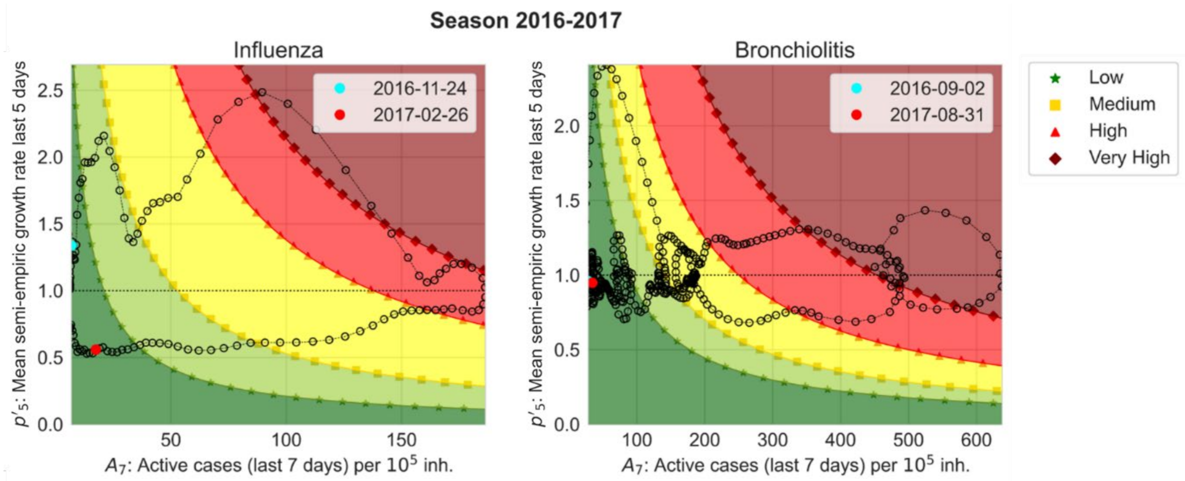

Supplementary Figure 9. Risk diagrams for season 2016-2017 for influenza (left) and bronchiolitis (right). They show ρ'_5 with respect to A_7 starting from the cyan point and finishing at the red point. The background colors correspond to EPG values classified by the epidemic levels. Very low (dark green) and low (light green) levels are separated by "*", low and medium (yellow) levels by a square, medium and high (red) levels by triangles and high and very high (maroon) levels by diamonds.

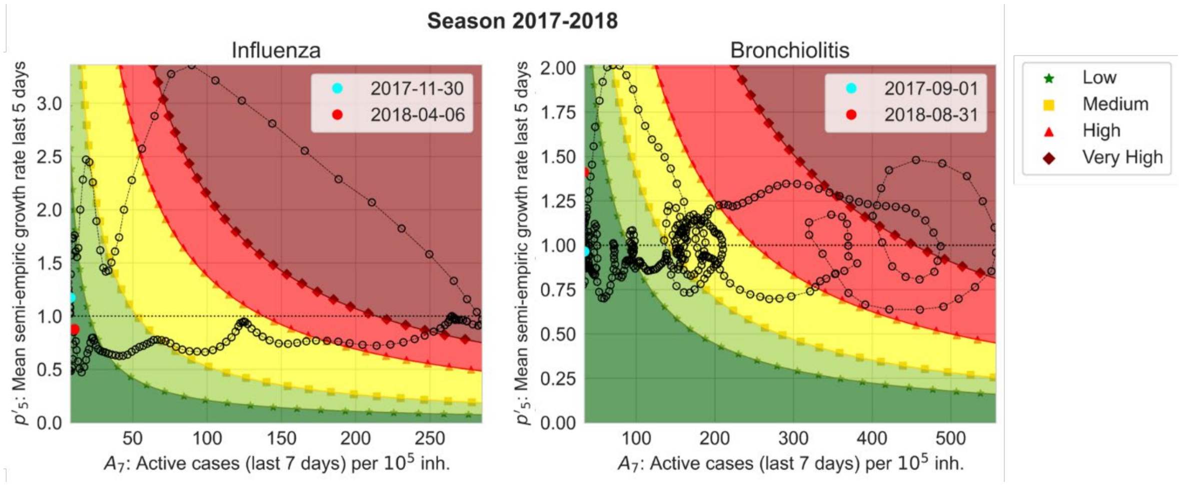

Supplementary Figure 10. Risk diagrams for season 2017-2018 for influenza (left) and bronchiolitis (right). They show ρ'_5 with respect to A_7 starting from the cyan point and finishing at the red point. The background colors correspond to EPG values classified by the epidemic levels. Very low (dark green) and low (light green) levels are separated by "*", low and medium (yellow) levels by a square, medium and high (red) levels by triangles and high and very high (maroon) levels by diamonds.

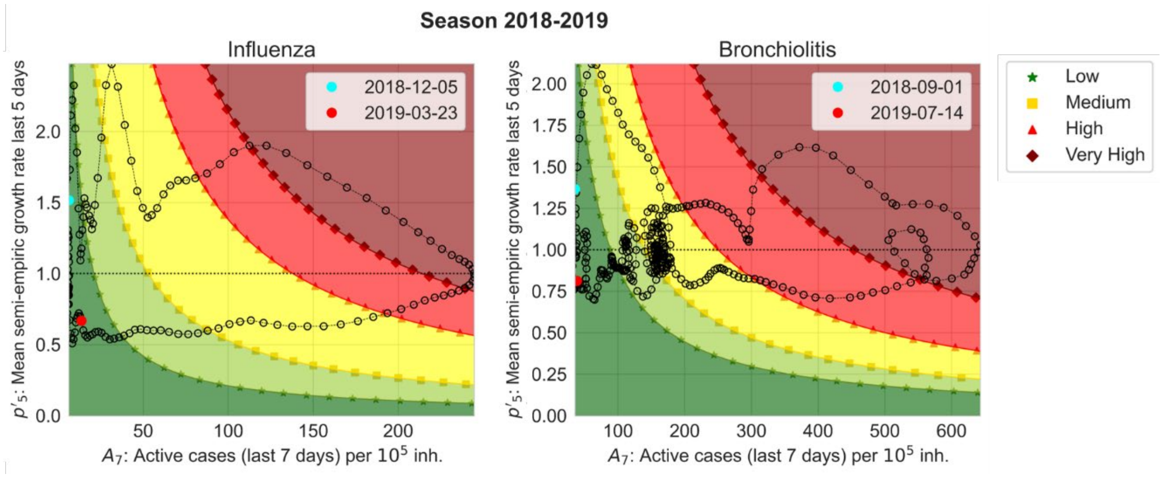

Supplementary Figure 11. Risk diagrams for season 2018-2019 for influenza (left) and bronchiolitis (right). They show ρ'_5 with respect to A_7 starting from the cyan point and finishing at the red point. The background colors correspond to EPG values classified by the epidemic levels. Very low (dark green) and low (light green) levels are separated by "*", low and medium (yellow) levels by a square, medium and high (red) levels by triangles and high and very high (maroon) levels by diamonds.

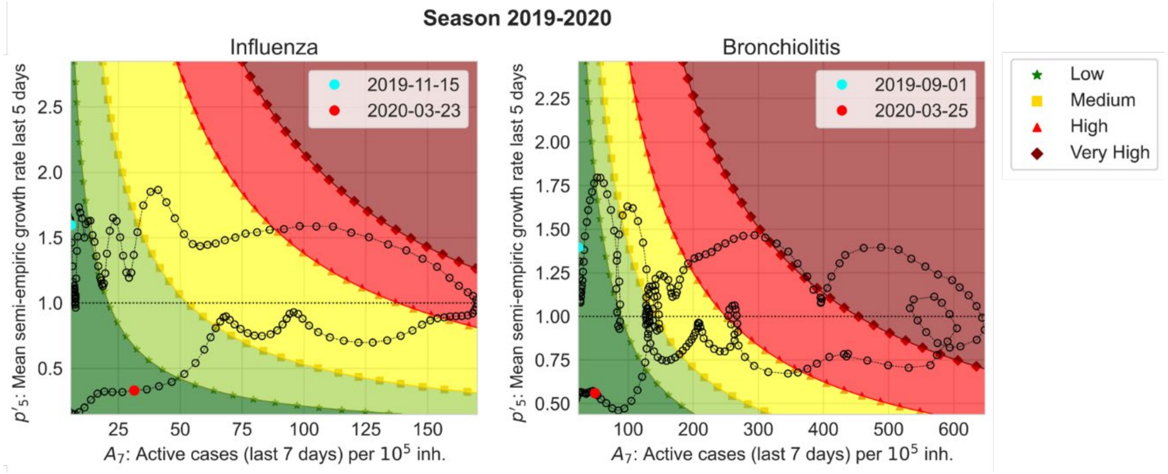

Supplementary Figure 12. Risk diagrams for season 2019-2020 for influenza (left) and bronchiolitis (right). They show ρ'_5 with respect to A_7 starting from the cyan point and finishing at the red point. The background colors correspond to EPG values classified by the epidemic levels. Very low (dark green) and low (light green) levels are separated by "*", low and medium (yellow) levels by a square, medium and high (red) levels by triangles and high and very high (maroon) levels by diamonds.

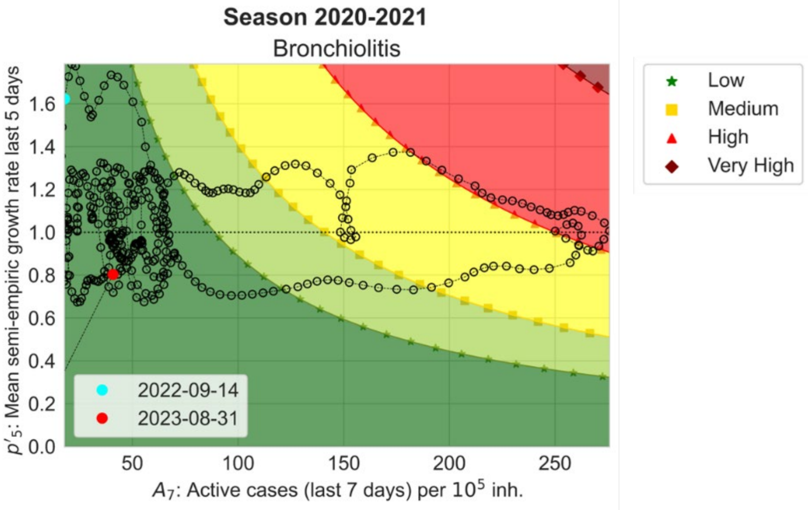

Supplementary Figure 13. Risk diagrams for season 2020-2021 for influenza (left) and bronchiolitis (right). They show ρ'_5 with respect to A_7 starting from the cyan point and finishing at the red point. The background colors correspond to EPG values classified by the epidemic levels. Very low (dark green) and low (light green) levels are separated by "*", low and medium (yellow) levels by a square, medium and high (red) levels by triangles and high and very high (maroon) levels by diamonds.

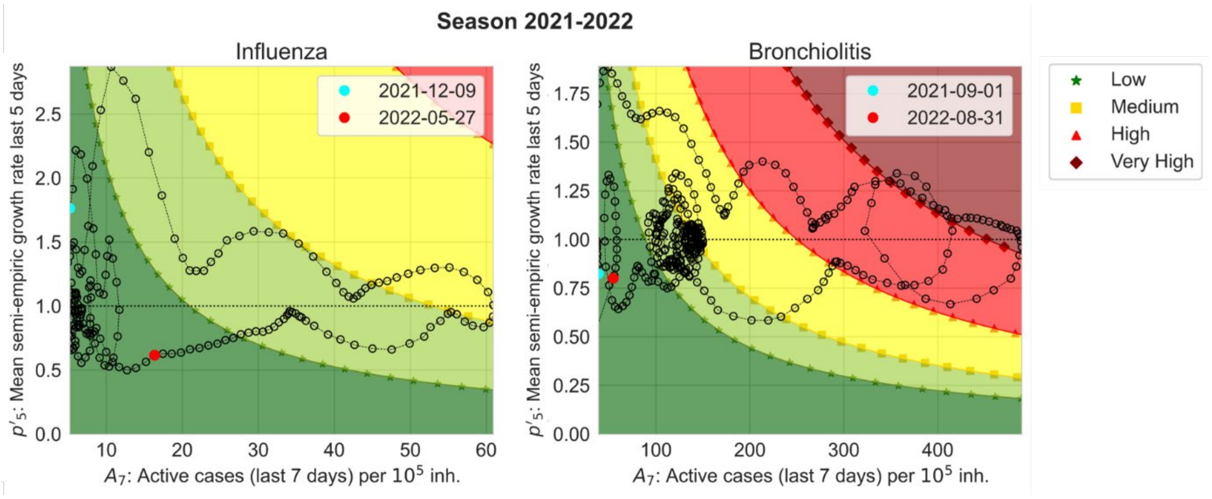

Supplementary Figure 14. Risk diagrams for season 2021-2022 for influenza (left) and bronchiolitis (right). They show ρ'_5 with respect to A_7 starting from the cyan point and finishing at the red point. The background colors correspond to EPG values classified by the epidemic levels. Very low (dark green) and low (light green) levels are separated by "*", low and medium (yellow) levels by a square, medium and high (red) levels by triangles and high and very high (maroon) levels by diamonds.

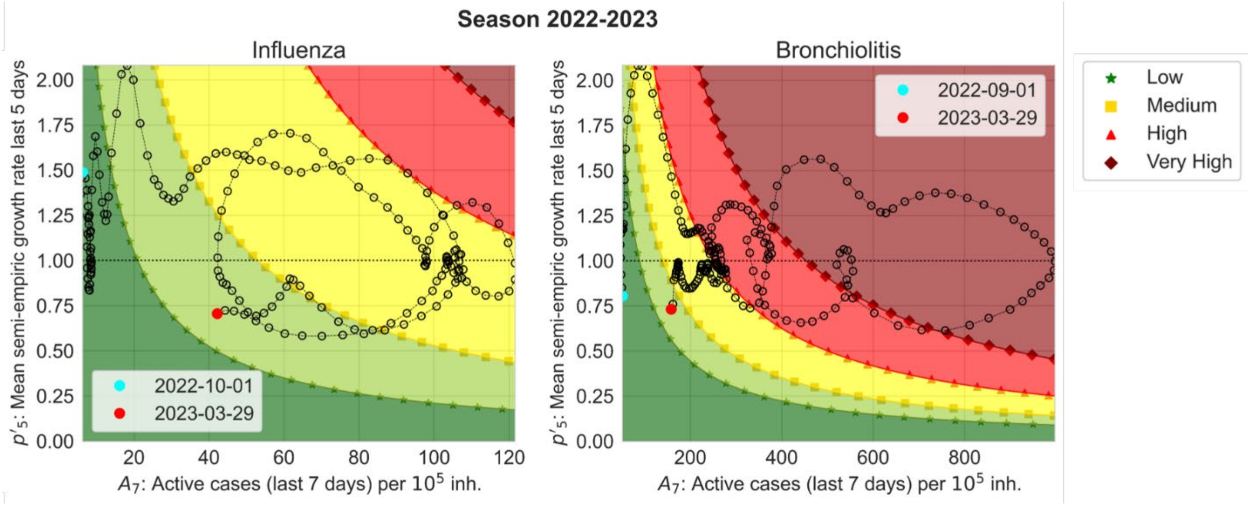

Supplementary Figure 15. Risk diagrams for season 2022-2023 for influenza (left) and bronchiolitis (right). They show ρ'_5 with respect to A_7 starting from the cyan point and finishing at the red point. The background colors correspond to EPG values classified by the epidemic levels. Very low (dark green) and low (light green) levels are separated by "*", low and medium (yellow) levels by a square, medium and high (red) levels by triangles and high and very high (maroon) levels by diamonds.
